# Seroprevalence of Immunoglobulin G antibodies against pertussis toxin among asymptomatic medical students in the west of Iran: a cross sectional study

**DOI:** 10.1186/1471-2334-9-58

**Published:** 2009-05-09

**Authors:** Seyyed Hamid Hashemi, Mitra Ranjbar, Mehrdad Hajilooi, Mohammad-Ali Seif-Rabiei, Mahnaz Bolandi, Javad Moghimi

**Affiliations:** 1 Depatment of Infectious Diseases, Hamedan University of Medical Sciences, Hamedan, Iran; 2 Depatment of Infectious Diseases, Iran University of Medical Sciences, Tehran, Iran; 3 Depatment of Immunology, Hamedan University of Medical Sciences, Hamedan, Iran; 4 Depatment of Social Medicine, Hamedan University of Medical Sciences, Hamedan, Iran

## Abstract

**Background:**

Pertussis is a highly communicable, vaccine-preventable respiratory infection. Immune response against this disease can be induced by infection or vaccination. Protection after childhood vaccination is minimal after ten years. Our aim was to assess pertussis immunity state in a population of healthy young medical students.

**Methods:**

In this seroepidemiological survey, blood samples were obtained from 163 first-year medical students in Hamedan University, Iran. Serum level of IgG against pertussis toxin (IgG-PT) was measured by Enzyme-Linked Immunosorbent Assay (ELISA) method. For qualitative assessment, IgG-PT levels more than 24 unit (U)/ml were considered positive. Data was analysed qualitatively and quantitatively considering gender and age groups.

**Results:**

There were 83 males and 80 females, with a mean age of 19.48 years, Prevalence of IgG-PT was 47.6% with mean level of 71.7 u/ml (95% confidence interval: 68.1–75.3). No statistically significant difference was observed with respect to sero-positivity of IgG-PT between males and females (45 cases (54%) vs. 34 cases (42%); P = 0.06). Mean IgG-PT levels in males and females were 84 U/ml and 58.8 U/ml, respectively (P = 0.91).

**Conclusion:**

A considerable proportion of our study population with a positive history of childhood vaccination for pertussis was not serologically immune to pertussis. A booster dose of acellular pertussis vaccine may be indicated in Iranian, medical students regarding their serologic conditions and outstanding role in health care systems.

## Background

Pertussis is a very communicable disease caused by *Bordetella pertussis *and all age groups are susceptible to this respiratory infection [1]. The incidence of adult pertussis has been estimated to be 200 to 500 per 100,000 person-years, even in highly immunised populations [2-4], which is thought to reflect waning of the immunity from childhood vaccination. A total number of 314 suspicious cases of pertussis, according to specimens collected by Dacron swabs from nasopharynx, were reported in Iran in 2007. Of this, 23 cases were diagnosed by pertussis using the laboratory method of Real Time polymerase chain reaction (PCR) [5]. Based on this report, the incidence of pertussis in Iran has decreased from 40% in 100,000 population in 1978 to 0.5% in 100,000 population in 2007.

Pertussis vaccines use in routine paediatric vaccination programs has dramatically decreased the incidence and complications of whooping cough in children [6], but protection is thought to be minimal after 10 years without boosting [7,8]. Because of concerns about the complications of whole-cell pertussis vaccines in older children and adults, booster vaccinations have not been recommended in these groups. In Iran, the pertussis vaccine is administered in the 2nd, 4th, and 6th months of life, in combination with two booster doses one administered in month 18 and the other between the years 4 to 6.

In spite of the worldwide decline in the infection incidence, the circulation of *B. pertussis *has not been eliminated [9]. During recent decades, numerous studies have documented that a significant percentage of prolonged cough illnesses among adolescents and adults are due to *B. pertussis*, with serological studies indicating a high rate of unrecognized infections [10-13]. These groups act as a source of infection for young infants who have not yet completed their primary immunisation [7,14]. Acellular pertussis vaccines have been evaluated in adolescents and adults and confer a safe and effective protection against pertussis in 92% of individuals [15]. However, universal adult booster vaccination against pertussis has remained controversial [7].

In this study, our aim was to determine the seroprevalence of *B. Pertussis *among a group of adolescent first-year medical students in the province of Hamedan, Iran in order to provide local epidemiological data. This study, together with other national studies, can supply evidence for recommending adult booster vaccination.

## Methods

This cross-sectional seroepidemiological study was conducted in healthy first-year medical students of Hamedan University, Iran yet did not have exposure to hospital environment. We considered the prevalence of pertussis seropositivity, difference and confidence interval as 30%, 0.07, and 95%, respectively. According to our calculations, a sample size of 163 subjects was selected by simple random method. Hence, all registration numbers of students were listed, and by applying table of random numbers, the subjects were selected. All individuals were asymptomatic while entering the study. Any respiratory disease or conditions affecting immunocompetence were of exclusion criteria. The subjects were classified into three age groups: less than 19 years, 19–21 years and over 21 years.. The data related to age, gender and vaccination status (according to their medical records) were obtained during a primary interview. All participants had received diphtheria, tetanus, whole pertussis vaccine (DTwP) manufactured by Razi Vaccine & Serum Research Institute, Tehran, Iran. Each dose of a o.5 ml of Razi-DTwP vaccine contains 15 Lf diphtheria toxoid, 10 lF tetanus toxoid, 16 IU inactivated Bordetella Perussis bacterial cells, 0.3 to 0.6 mg aluminium phosphate (metal iron) and 0.01% merthiolate according to thr instruction sheet provided by the manufacturer [16].

For serological assessment, a venous blood sample of 3–5 ml was drawn. Immunoglobulin G (IgG) antibodies against pertussis toxin, IgG-PT, were measured by Enzyme-Linked Immunosorbent Assay (ELISA) kits and results were interpreted according to the instructions provided by the manufacturer (IBL Immuno-Biological Laboratories, Hamburg, Germany). For qualitative assessment, IgG antibody levels more than 24 units (U)/ml were considered positive. Results were also analysed regarding quantitative values.

Age and sex distribution at enrolment, the total prevalence of seropositive results and mean level of pertussis IgG-PT in the studied subjects were calculated. In addition, the findings were stratified by the age groups. Difference in sero-positivity percentages was calculated using the chi-square test. Quantitatively, t-test or one-way ANOVA was used for examining mean IgG-PT levels between the subgroups. Significance level was set at p < 0.05.

The study was approved by the Ethics Committee of our university and informed consent was obtained from all study participants.

## Results and discussion

A total of 163 individuals (83 males and 80 females) were recruited between March and June 2007. Mean age of the cases was 19.48 years, ranging from 16 to 24 years. Of these, 112 subjects were under 19 years, 40 were 19–21 years, and 11 were above 21 years old. History of pertussis vaccination was positive in all subjects.

The total sero-prevalence rate of IgG-PT was 47.6%, with a mean level of 71.7 U/ml (95% Confidence Interval: 68.1–75.3). Table 1 shows the frequency of IgG-PT positivity in the studied population. As presented, there were no statistically significant differences neither regarding the frequency of sero-positivity between males and females nor between the age groups.

**Table 1:** The frequency of anti-PT seropositivity according to age and gender.

Parameters(total)	N	%	p-value
Sex			
Male (83)	45	54	0.06
Female (80)	34	42	
Age group			
<19 (112)	37	33	0.09
19–21 (40)	20	51	
>21 (11)	5	45	

Mean IgG-PT levels in males and females were 84 U/ml and 58.8 U/ml, respectively (P = 0.91). In the analysis performed regarding the age groups, mean IgG-PT level was 37 U/ml for <19 years, 82.7 U/ml for 19–21 years, and 49.2 U/ml for more than 21 year-old subjects. There were no significant differences in the mean IgG-PT levels among the age subgroups (p = 0.06).

According to obtained results, a considerable number of medical students studied in an Iranian university from both genders were not serologically immune to pertussis. Since these students will have a major role in providing health care services in both in-patient and out-patient medical centres in the near future, it is of high priority to perform complete vaccination programs through acellular pertussis vaccines.

The importance of determination of IgG-PT in student populations has been implicated in former studies. In a study performed on students with chronic cough at the University of California in 1992, 34 cases (26%) of infection with pertussis were found [12]. This led to this suggestion that *B. pertussis *cough illnesses were endemic in adolescents and adults and that they could be considered as a source of outbreaks of the disease in susceptible children. Subsequently, a number of studies among symptomatic and cough-free adolescents and adults have noted a high percentage of the illnesses due to *B. Pertussis *infection [10,11].

We documented 47.6% seropositivity of IgG-PT in the asymptomatic adolescent population between the ages of 19 and 24 years with a similar distribution between men and women. According to national childhood vaccination programme in Iran, all of our subjects had complete history of immunization against pertussis and none of them received any adult booster vaccine dose. Our data is consistent with another study in Iran (Isfahan) which reported 48% positive serology for IgG-PT in adolescents with prolonged cough [17]. The current statistics are also in agreemenr with those from some other countries [18,19].

In previous serological studies performed in different countries, pertussis protective antibodies were reported in a wide range of 30 to 97 percent. Vaccine antibodies begin to wane 4 years after the last dose, thus immunity to pertussis vaccine diminishes to 0% to 20% over a 10-year interval [7,8]. It has been shown that the seroprevalence increases with age especially in countries with good childhood vaccination coverage [10-13]. The increment between the adolescent and adult age group is assumed to be due to natural pertussis infection. Repeated exposure to the infection can also explain high level of pertussis antibody among unvaccinated adults.

Apart from being an adolescent population, our study subjects were medical students. Health care workers have more professional contacts with infectious patients than general population. As our finding showed, half of the studied population were seronegative. Therefore, This group will be more susceptible to pertussis in the future working as primary care providers in hospitals. Previous outbreaks among healthcare workers and their patients can signify the potentially harmful consequence of such results [20-22]. However, it should be considered that protection against B. pertussis is multifactorial. The presence of circulating antibodies does not guarantee the protection and the absence of antibodies does not imply that an individual is necessarily susceptible.

Acellular pertussis vaccines confer safe and effective protection against pertussis in adolescents and adults [23]. In March 2006, the American Academy of Paediatrics published their recommendation that adolescents 11 to 18 years of age should receive a single dose of acellular pertussis vaccines combined with diphtheria and tetanus toxoids (DTaP) instead of tetanus and diphtheria toxoids vaccine for booster immunisation.

In this study, only medical students were included. Therefore, the obtained results may not be extended to adolescents and adults living in the studied region. Also, we did not follow these students in order to document the clinical and serologic consequences of administration of a booster dose of DTaP. Although all studied individuals were students at the first year of medical school passing their basic courses, we were not able to establish any documented contact with B. Pertussis.

Further studies are required to determine the incidence, morbidity and complications of pertussis in adolescents and adults to evaluate the cost-effectiveness of vaccination.

## Conclusion

A considerable proportion of the study population with a positive history of childhood vaccination for pertussis was not serologically immune to pertussis. A booster dose of acellular pertussis vaccine may be indicated in Iranian, medical students regarding their serologic conditions and outstanding role in health care systems.

## Abbreviations

DTaP: diphtheria and tetanus toxoids; ELISA: Enzyme-Linked Immunosorbent Assay; IgG-PT: IgG against pertussis toxin.

## Competing interests

The authors declare that they have no competing interests.

## Authors' contributions

SHH, MR and JM planned the study design and drafted and edited the manuscript. MH carried out the immunoassays. MASR participated in data collection and conceived the study. MB participated in the study design and performed the statistical analysis.

All authors read and approved the final manuscript.

## Pre-publication history

The pre-publication history for this paper can be accessed here:

http://www.biomedcentral.com/1471-2334/9/58/prepub
